# Comparison of Early Warning Scoring Systems for Hospitalized Patients With and Without Infection at Risk for In-Hospital Mortality and Transfer to the Intensive Care Unit

**DOI:** 10.1001/jamanetworkopen.2020.5191

**Published:** 2020-05-19

**Authors:** Vincent X. Liu, Yun Lu, Kyle A. Carey, Emily R. Gilbert, Majid Afshar, Mary Akel, Nirav S. Shah, John Dolan, Christopher Winslow, Patricia Kipnis, Dana P. Edelson, Gabriel J. Escobar, Matthew M. Churpek

**Affiliations:** 1 Division of Research, Kaiser Permanente Northern California, Oakland; 2 Department of Medicine, University of Chicago, Chicago, Illinois; 3 Department of Medicine, Loyola University Medical Center, Chicago, Illinois; 4 NorthShore University HealthSystem, Evanston, Illinois

## Abstract

**Importance:**

Risk scores used in early warning systems exist for general inpatients and patients with suspected infection outside the intensive care unit (ICU), but their relative performance is incompletely characterized.

**Objective:**

To compare the performance of tools used to determine points-based risk scores among all hospitalized patients, including those with and without suspected infection, for identifying those at risk for death and/or ICU transfer.

**Design, Setting, and Participants:**

In a cohort design, a retrospective analysis of prospectively collected data was conducted in 21 California and 7 Illinois hospitals between 2006 and 2018 among adult inpatients outside the ICU using points-based scores from 5 commonly used tools: National Early Warning Score (NEWS), Modified Early Warning Score (MEWS), Between the Flags (BTF), Quick Sequential Sepsis-Related Organ Failure Assessment (qSOFA), and Systemic Inflammatory Response Syndrome (SIRS). Data analysis was conducted from February 2019 to January 2020.

**Main Outcomes and Measures:**

Risk model discrimination was assessed in each state for predicting in-hospital mortality and the combined outcome of ICU transfer or mortality with area under the receiver operating characteristic curves (AUCs). Stratified analyses were also conducted based on suspected infection.

**Results:**

The study included 773 477 hospitalized patients in California (mean [SD] age, 65.1 [17.6] years; 416 605 women [53.9%]) and 713 786 hospitalized patients in Illinois (mean [SD] age, 61.3 [19.9] years; 384 830 women [53.9%]). The NEWS exhibited the highest discrimination for mortality (AUC, 0.87; 95% CI, 0.87-0.87 in California vs AUC, 0.86; 95% CI, 0.85-0.86 in Illinois), followed by the MEWS (AUC, 0.83; 95% CI, 0.83-0.84 in California vs AUC, 0.84; 95% CI, 0.84-0.85 in Illinois), qSOFA (AUC, 0.78; 95% CI, 0.78-0.79 in California vs AUC, 0.78; 95% CI, 0.77-0.78 in Illinois), SIRS (AUC, 0.76; 95% CI, 0.76-0.76 in California vs AUC, 0.76; 95% CI, 0.75-0.76 in Illinois), and BTF (AUC, 0.73; 95% CI, 0.73-0.73 in California vs AUC, 0.74; 95% CI, 0.73-0.74 in Illinois). At specific decision thresholds, the NEWS outperformed the SIRS and qSOFA at all 28 hospitals either by reducing the percentage of at-risk patients who need to be screened by 5% to 20% or increasing the percentage of adverse outcomes identified by 3% to 25%.

**Conclusions and Relevance:**

In all hospitalized patients evaluated in this study, including those meeting criteria for suspected infection, the NEWS appeared to display the highest discrimination. Our results suggest that, among commonly used points-based scoring systems, determining the NEWS for inpatient risk stratification could identify patients with and without infection at high risk of mortality.

## Key Points

**Question:**

How do common tools using points-based risk scores compare for identifying high-risk hospitalized inpatients with and without infection?

**Findings:**

In this cohort study of 5 commonly used point-based risk scores for 1.5 million hospitalizations across 2 US states, the National Early Warning Score had the highest discrimination for identifying inpatients at risk for death and/or intensive care unit transfer. Compared with other tools calculating risk scores, the National Early Warning Score was also more efficient at all sensitivity thresholds.

**Meaning:**

These results suggest that, among common points-based scoring systems, using the National Early Warning Score for inpatient risk stratification could identify patients with and without infection at high risk of mortality.

## Teaser

This cohort study examines 5 scoring systems used to estimate the risk of clinical deterioration in hospitalized patients with and without suspected infection.

## Introduction

Clinical deterioration leading to death or intensive care unit (ICU) transfer affects 3% to 5% of patients hospitalized outside the ICU and is associated with increased morbidity and mortality.^1,2^ These increased risks are particularly heightened among patients with serious infection or sepsis, which contributes to 50% or more of hospital deaths.^3^ Previous studies have shown that many patients exhibit signs of increased risk hours before deterioration and that these early signals can be captured by routinely measured clinical data, such as vital signs or laboratory test results.^1,2,4^ This finding has led researchers, professional societies, and health systems to develop and implement risk scores within early warning systems that use routinely available clinical data and can alert clinicians to intervene in patients at risk for impending deterioration.^1,2,5,6^ Several groups, including the Joint Commission, the Centers for Medicare & Medicaid Services, and the Institute for Healthcare Improvement, have further promoted regulations and guidelines that have heightened the focus on leveraging risk scores to accelerate the identification and treatment of patients with deteriorating and septic status.^7,8,9,10^

While many risk score tools exist, variability in their reported performance has led to uncertainty about how these scoring systems compare with one another. Most previous work has been performed at single centers and has used a small number of tools.^11,12,13^ Furthermore, it remains unclear whether a general risk score developed in an undifferentiated inpatient population will display similar performance as that of scores determined through systems specifically targeted to patients with suspected infection. Because there is considerable overlap between these scoring systems, the targeted at-risk populations, and the clinical staff responding to alerts, an approach that uses a single risk score for screening has the potential to reduce the alarm burden, improve the efficiency of clinical and technical training and implementation, all of which are factors in earlier recognition and effective treatment for patients whose condition is deteriorating.

In this study, we sought to compare the performance of 5 tools used to determine risk scores that are often incorporated within early warning systems for predicting the risk of clinical deterioration among inpatients outside the ICU across 28 hospitals in California and Illinois. We then assessed how risk score performance differed when inpatients were stratified by the presence or absence of suspected infection.

## Methods

We identified adults (aged ≥18 years) hospitalized at 22 Kaiser Permanente Northern California hospitals from 2010 to 2015 and 6 Illinois hospitals (University of Chicago [2008-2018], Loyola University Medical Center [2007-2017], and 4 hospitals in the NorthShore University Health System [2006-2016]). We included inpatients with at least 1 vital sign measured in the emergency department (ED) or a non-ICU hospital ward. We excluded patients who died in the ED before inpatient admission, were discharged directly from the ED, were admitted directly to the ICU, or were in labor and delivery wards. We evaluated comorbid disease prevalence based on the Elixhauser index.^14^ Data analysis was conducted from February 2019 to January 2020.

This study was approved by the Kaiser Permanente Northern California, University of Chicago, Loyola University Medical Center, and NorthShore University HealthSystem institutional review boards with a waiver of informed consent. This study followed the Strengthening the Reporting of Observational Studies in Epidemiology (STROBE) reporting guideline for cohort studies.

We defined clinical deterioration based on 2 outcomes common to evaluating risk score performance: hospital death and the combined outcome of ICU transfer or death. For each hospitalization, we defined a patient’s time-at-risk interval as the hospital period preceding either outcome and, if there were more than one at-risk interval, used clinical data from only the first time-at-risk interval to calculate maximum score values.^12^

We identified patients with suspected infection based on the dyad criteria established in the Sepsis-3 definitions (ie, paired time intervals between an order for microbiologic culture and antibiotic prescription) with the timestamp of the first dyad element marking the onset of suspected infection.^15^ If the onset of suspected infection occurred during the time-at-risk interval, the patient’s hospitalization was deemed suspected infection; otherwise, the hospitalization was deemed not suspected infection.

We evaluated 5 points-based risk score tools often used within early warning system programs to identify high-risk patients. We did not evaluate machine learning–based algorithms because they are not generalizable to many health care settings, including those without electronic health record (EHR) systems and/or with limited resources. The general scoring systems included National Early Warning Score (NEWS),^16^ Modified Early Warning Score (MEWS),^17^ and Between the Flags (BTF) criteria.^18^ The infection-targeted scoring systems were Quick Sequential Sepsis-Related Organ Failure Assessment (qSOFA)^15^ and Systemic Inflammatory Response Syndrome (SIRS) criteria.^19^ eTable 1 in the Supplement displays each tool’s data elements.

Using EHR data, we calculated each risk score using previously described approaches.^1,2,12^ The goal was to define the single maximum value of each score achieved by a patient during their time-at-risk interval.^12^ Thus, starting with a patient’s first documented vital sign, the scores were recalculated with each new data element during the interval using imputation to normal for missing values at the outset and a last-value-carried-forward approach for other values.^4^ eTable 2 in the Supplement displays the missingness rates for data elements, which ranged from less than 1% for vital signs to 87% for abnormal band forms of white blood cells of greater than 10%. This calculation strategy was used to approximate how these scores would be calculated when applied in clinical practice. We then used the maximum value during the time-at-risk interval to evaluate whether a patient’s condition would have crossed specific alerting thresholds.

### Statistical Analysis

Data are described using number (frequency), mean (SD), and median (interquartile range [IQR]). We assessed model discrimination in each state (California and Illinois) using area under the receiver operating characteristic curves (AUCs) and 95% CIs based on the maximum score value for each outcome. We further evaluated discrimination in the overall population and subgroups stratified by suspected infection.

We generated risk score efficiency curves for in-hospital mortality, which display the percentage of the at-risk cohort that would need to be screened to achieve various sensitivity thresholds. We evaluated score cut points associated with frequently used thresholds for SIRS (≥2) and qSOFA (≥2). In post hoc analysis, we also compared performance at NEWS thresholds that demonstrate similarly high sensitivity for hospital mortality with SIRS greater than or equal to 2 (NEWS≥6) and high specificity to qSOFA greater than or equal to 2 (NEWS≥8). In sensitivity analyses, we evaluated the ordering of model discrimination when (1) using only hospitalizations with complete score data, (2) excluding data from the final hour before the outcome, and (3) including only a single hospitalization for patients with multiple hospitalizations. Differences were considered significant at *P* < .05. Data analysis was performed with Stata/SE, version 14.2, Stata/MP, version 15.1 (StataCorp), and SAS, version 9.4 (SAS Institute Inc).

## Results

The study included a total of 1 487 263 hospitalizations, with approximately equal distribution from the California hospitals (n = 773 477; mean [SD] age, 65.1 [17.6] years; 416 605 women [53.9%]) and Illinois hospitals (n = 713 786; mean [SD] age, 61.3 [19.9] years; 384 830 women [53.9%]) (Table). Across both states, 484 125 patients (32.6%) met the suspicion of infection criteria. Compared with patients without suspected infection, patients with suspected infection were older (mean [SD] age, 68.0 [17.6] years in California and 62.9 [18.2] years in Illinois vs 63.3 [17.3] years in California and 60.7 [17.8] years in Illinois) and more frequently hospitalized through the ED (261 579 [91.0%] vs 283 146 [58.3%] in California and 125 649 [63.9%] vs 243 011 [47.0%] in Illinois). Mortality was 2.9% (n = 22 786) in the California cohort and 1.6% (n = 11 250) in the Illinois cohort; 13.9% (n = 107 776) of the California cohort and 9.7% (n = 69 320) of the Illinois cohort experienced the outcome of ICU transfer or death.

**Table.** Baseline Characteristics of Hospital Encounters Stratified by State and Suspected Infection

Characteristic	Suspected infection, No. (%)			
	California hospitals (n = 773 477)		Illinois hospitals (n = 713 786)	
	Yes	No	Yes	No
No. of patients	287 510 (37.1)	485 967 (62.8)	196 615 (27.5)	517 171 (72.5)
Men	131 583 (45.8)	225 289 (46.4)	88 928 (45.2)	240 028 (46.4)
Age, mean (SD)	68.0 (17.6)	63.3 (17.3)	62.9 (18.2)	60.7 (17.8)
Admitted via ED	261 579 (91.0)	283 146 (58.3)	125 649 (63.9)	243 011 (47.0)
Length of stay, median (range), d	4 (2-6)	2 (1-4)	4 (3-8)	2 (1-4)
Hospital death	15 685 (5.5)	7101 (1.5)	5889 (3.0)	5361 (1.0)
ED to ICU admission	33 630 (11.7)	25 550 (5.3)	12 890 (6.6)	29 073 (5.6)
Selected Elixhauser comorbidities				
Congestive heart failure	84 255 (29.3)	92 754 (19.1)	47 502 (24.2)	94 961 (18.4)
Valvular disease	49 175 (17.1)	62 718 (12.9)	34 173 (17.4)	78 185 (15.1)
Pulmonary circulation disorders	29 140 (10.1)	29 113 (6)	16 058 (8.2)	29 181 (5.6)
Peripheral vascular disorders	113 540 (39.5)	135 190 (27.8)	33 422 (17.0)	72 240 (14.0)
Uncomplicated hypertension	215 143 (74.8)	322 233 (66.3)	124 601 (63.4)	301 983 (58.4)
Paralysis	31 559 (11.0)	29 377 (6.0)	10 159 (5.2)	15 907 (3.1)
Neurodegenerative disorders	59 166 (20.6)	60 942 (12.5)	29 556 (15.0)	58 048 (11.2)
Chronic pulmonary disease	118 352 (41.2)	163 846 (33.7)	43 840 (22.3)	85 408 (16.5)
Diabetes, uncomplicated	110 139 (38.3)	140 783 (29)	61 099 (31.1)	131 439 (25.4)
Hypothyroidism	56 452 (19.6)	78 612 (16.2)	30 332 (15.4)	70 063 (13.5)
Renal failure	104 052 (36.2)	120 197 (24.7)	45 754 (23.3)	77 393 (15)
Liver disease	30 394 (10.6)	42 934 (8.8)	21 205 (10.8)	40 417 (7.8)
Peptic ulcer disease	1761 (0.6)	2121 (0.4)	1932 (1.0)	3468 (0.7)
Metastatic cancer	25 918 (9)	31 866 (6.6)	19 620 (10.0)	38 727 (7.5)
Solid tumor	64 138 (22.3)	101 011 (20.8)	41 267 (21.0)	97 285 (18.8)
Autoimmune disease	24 062 (8.4)	29 763 (6.1)	12 711 (6.5)	25 525 (4.9)
Coagulopathy	47 690 (16.6)	49 293 (10.1)	37 882 (19.3)	55 810 (10.8)
Fluid/electrolyte disorders	158 282 (55.1)	176 364 (36.3)	89 478 (45.5)	148 614 (28.7)
Blood loss anemia	21 712 (7.6)	26 793 (5.5)	9561 (4.9)	18 241 (3.5)
Iron deficiency anemia	147 251 (51.2)	174 853 (36)	13 833 (7)	22 647 (4.4)
Depression	112 412 (39.1)	164 951 (33.9)	40 431 (20.6)	86 294 (16.7)

Abbreviations: ED, emergency department; ICU, intensive care unit.

Maximum values of the 5 risk score tools outside the ICU had similar distributions across the 2 states, both in the overall hospitalized cohort and in suspected infection subgroups (eTable 3 in the Supplement). For example, the median maximum value of the NEWS among the suspected infection subgroup was 6 (IQR, 4-8) in both states. In the California sample, the median number of elapsed hours between first reaching risk score thresholds and the composite outcome of ICU transfer or death was 5.3 (IQR, 2.7-27.3) hours for a qSOFA score greater than or equal to 2, 6.1 (IQR, 3.3-28.6) hours for a SIRS score greater than or equal to 2, and 5.7 (IQR, 3.1-25.3) hours for a NEWS greater than or equal to 6. Intervals that were somewhat longer were observed in the Illinois sample of 8.2 (IQR, 3.3-34.6) hours for a qSOFA score greater than or equal to 2, 9.5 (IQR, 4.1-42.1) hours for a SIRS score greater than or equal to 2, and 9.2 (IQR, 3,8-40.9) hours for a NEWS greater than or equal to 6.

**Figure 1.**
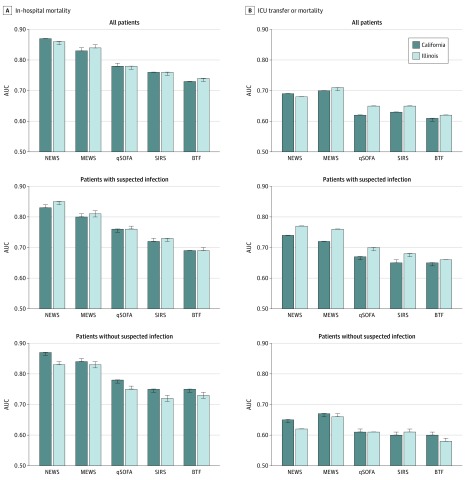
Discrimination of Risk Scores for In-Hospital Mortality and the Combined Outcome of Intensive Care Unit (ICU) Transfer or Mortality Area under the receiver operating characteristic curve (AUC) for in-hospital mortality (A) and the combined outcome of ICU transfer or mortality (B) for all patients, patients with suspected infection, and patients without suspected infection. BTF indicates Between the Flags; MEWS, Modified Early Warning Score; NEWS, National Early Warning Score; qSOFA, Quick Sepsis-Related Organ Failure Assessment; and SIRS, Systemic Inflammatory Response Syndrome. Error bars indicate 95% CIs, and values without error bars indicate that the minimum and maximum error values equaled the point estimate.

In the entire hospitalized cohort (Figure 1A), model discrimination for death was highest for NEWS across both states (AUC, 0.87; 95% CI, 0.87-0.87 in California vs AUC, 0.86; 95% CI, 0.85-0.86 in Illinois), followed by MEWS (AUC, 0.83; 95% CI, 0.83-0.84 in California vs AUC, 0.84; 95% CI, 0.84-0.85 in Illinois). The NEWS and MEWS tools also showed the highest discrimination for the combined outcome of ICU transfer or death (Figure 1B). In the overall cohort, qSOFA and SIRS showed similar discrimination, but the scores obtained with both tools were lower than with the NEWS and MEWS. For example, AUC values for death as a separate variable were 0.78 (95% CI, 0.78-0.79) in California and 0.78 (95% CI, 0.77-0.78) in Illinois for qSOFA and 0.76 (95% CI, 0.76-0.76) in California and 0.76 (95% CI, 0.75-0.76) in Illinois for SIRS. The BTF tool had the lowest discrimination in the overall cohort, with an AUC for death of 0.73 (95% CI, 0.73-0.73) in the California hospitals and 0.74 (95% CI, 0.73-0.74) in the Illinois hospitals.

A similar pattern was seen in the suspected infection cohort (Figure 1), with the NEWS demonstrating the highest AUC for both outcomes across both states, followed by the MEWS. Even among patients with infection, the discrimination of the NEWS and MEWS were higher in all cases than the infection-specific risk scores. Similar patterns were demonstrated among the subgroup of patients without suspected infection for both outcomes (Figure 1), although all risk scores showed poor discrimination for the combined outcome of ICU transfer or death. For example, the AUCs were 0.65 (95% CI, 0.64-0.65) for California and 0.62 (95% CI, 0.62-0.62) for Illinois with the NEWS and 0.67 (95% CI, 0.66-0.67) for California and 0.66 (95% CI, 0.66-0.67) for Illinois with the MEWS.

**Figure 2.**
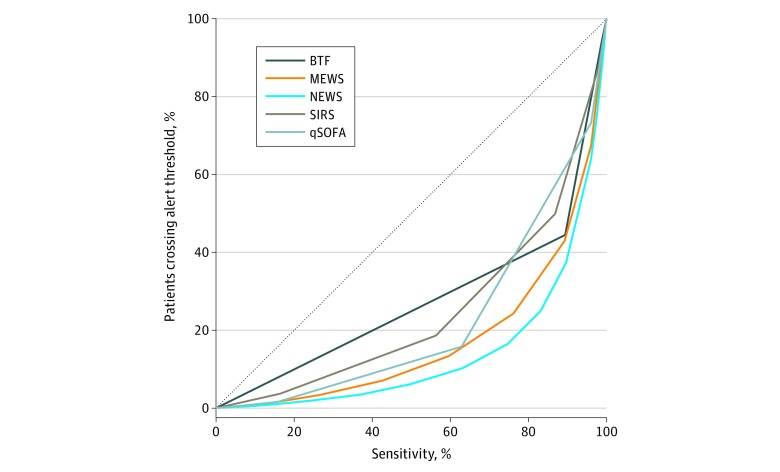
Early Warning Score Efficiency Curves for In-Hospital Mortality in All Patients Proportion of patients who reach each score threshold against the risk score sensitivity in evaluation of in-hospital mortality. The National Early Warning Score (NEWS) was associated with the lowest percentage of patients who crossed the alert threshold and would require screening. BTF indicates Between the Flags; MEWS, Modified Early Warning Score; qSOFA, Quick Sepsis-Related Organ Failure Assessment; and SIRS, Systemic Inflammatory Response Syndrome.

**Figure 3.**
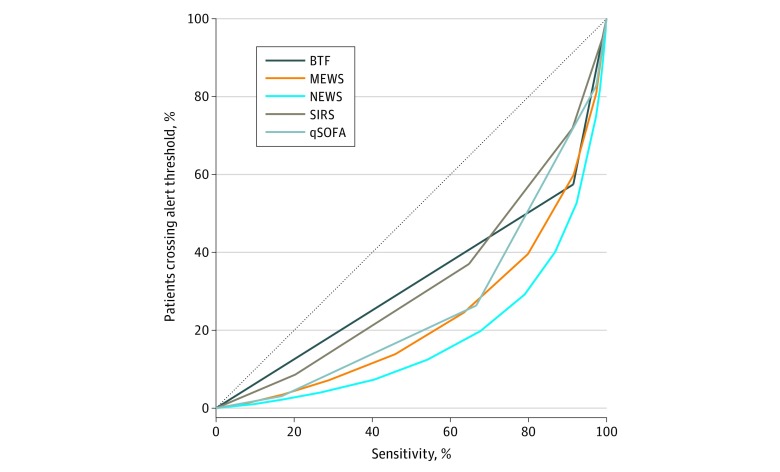
Early Warning Score Efficiency Curves for Patients With Suspected Infection Proportion of patients who reach each score threshold against the risk score sensitivity for suspected infection. Across any sensitivity threshold, the National Early Warning Score (NEWS) was associated with the lowest percentage of patients who crossed the alert threshold and would require screening. BTF indicates Between the Flags; MEWS, Modified Early Warning Score; qSOFA, Quick Sepsis-Related Organ Failure Assessment; and SIRS, Systemic Inflammatory Response Syndrome.

Figure 2 depicts a risk score efficiency curve among all hospitalized patients showing that, across all sensitivity thresholds for hospital mortality, using the NEWS would result in the fewest patients exceeding the alert threshold and requiring screening. For example, at a similar high-sensitivity threshold to a SIRS score greater than or equal to 2, a NEWS cutoff greater than or equal to 6 was both more sensitive (NEWS, 87%-89% vs SIRS, 86%-87% across both states) and resulted in fewer patients needing to be screened (NEWS, 36%-37% vs SIRS, 50% across both states). At a similar high-specificity threshold to qSOFA score greater than or equal to 2, a NEWS greater than or equal to 8 threshold had a higher sensitivity than qSOFA score greater than or equal to 2 (NEWS, 72%-74% vs qSOFA, 59%-63% across both states) and required a similar percentage of the population to be screened (NEWS, 16% for both states vs qSOFA, 13%-16% across both states). Findings were similar in patients with suspected infection (Figure 3).

**Figure 4.**
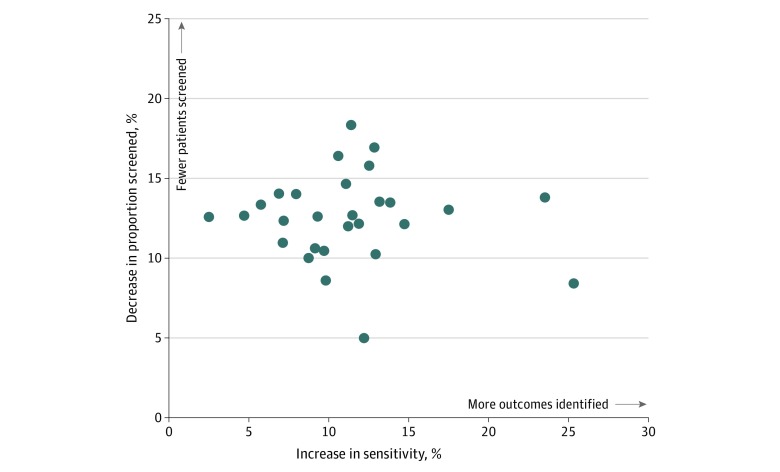
Reduction in Clinical Workload With National Early Warning Score (NEWS) vs Systemic Inflammatory Response Syndrome (SIRS) and Quick Sepsis-Related Organ Failure Assessment (qSOFA) Scoring Systems Each point represents one of the study hospitals and displays the estimated decrease in the proportion of patients screened at a similar sensitivity threshold (y-axis) and the increase in sensitivity as a similar specificity threshold (x-axis).

In the overall cohort, at a similar screening workload to the high-specificity qSOFA score greater than or equal to 2, a NEWS greater than or equal to 8 would have identified 4099 more patients who died overall and 2636 more in the suspected infection cohort than the qSOFA score. At a similar sensitivity threshold to the high-sensitivity SIRS score greater than or equal to 2, a NEWS greater than or equal to 6 would have required screening 200 325 fewer patients overall and 97 595 fewer patients with suspected infection than the SIRS. Figure 4 displays the relative reduction in clinical workload that would be associated with using a high-specificity NEWS greater than or equal to 8 vs a qSOFA score greater than or equal to 2 threshold or a high-sensitivity NEWS greater than or equal to 6 vs a SIRS score greater than or equal to 2 threshold. At each study hospital, the NEWS scores increased the identification of patients who experienced adverse outcomes or reduced the number of patients needing to be screened.

## Discussion

In this study, we compared the performance of 5 commonly used tools that use points-based risk scores to trigger early warning systems among 1.5 million hospitalizations from 28 hospitals in several health systems across 2 US states. We found that the general risk NEWS showed better discrimination for hospital mortality than the other tools in the overall hospitalized cohort and in subgroups stratified by suspected infection. Model discrimination for the combined outcome of ICU transfer or death was lower in all scenarios, with the NEWS and MEWS showing similar performance. This result appeared to be associated with poor discrimination in the noninfected subgroups. In all scenarios, NEWS outperformed infection-targeted scores, such as those calculated with qSOFA and SIRS, and may offer the opportunity to provide a single points-based score with efficiency characteristics suitable for identifying high-risk patients with and without infection.

Our findings have several implications. First, the results suggest that, for the goal of detecting clinical deterioration in hospitalized, non-ICU patients, aggregate weighted risk scores, such as those determined with NEWS and MEWS, outperform infection-specific scores, even among patients with suspected infection. This finding is consistent with previous single-center studies in patients with suspected infection that have evaluated patients in different countries and in specific hospital settings, although other small studies have suggested that NEWS and MEWS had only fair to poor accuracy in patients with sepsis.^5,11,20,21^ SIRS was not developed as an early warning score, but was designed to screen for and define sepsis, and SIRS criteria include parameters that are known to have limited predictive power for clinical deterioration.^19^ However, previous studies and existing programs have used the results of SIRS and qSOFA as triggers for early warning systems to screen for high-risk patients with infection. While the NEWS and MEWS are somewhat more complex to calculate than the SIRS and qSOFA scores, the NEWS is already used as a clinician-calculated score to risk-stratify large populations of acutely ill patients in the UK’s National Health Service.^22^ Our evidence suggests that the NEWS represents the most efficient choice among these commonly used tools for risk stratifying inpatients with suspected infection. In these scenarios, NEWS alert thresholds of 6 (higher sensitivity) and 8 (higher specificity) may be able to improve efficiency compared with SIRS or qSOFA score thresholds greater than or equal to 2.

Second, our findings also make the NEWS an efficient choice to fill the role of an all-purpose, points-based risk stratification tool for hospital mortality for all non-ICU inpatients. While smaller studies have shown possible validation of these scores across a variety of settings and populations, to our knowledge, our study is the largest multicenter investigation to date to evaluate these findings in a diverse ED and inpatient population.^5,11,12,16,17,20,21,22^ Given that NEWS includes all of the variables from qSOFA and BTF, patients identified as higher risk by scores from these other tools will similarly have elevated NEWS values. Our findings also appear to support work suggesting that aggregate weighted scores, such as NEWS, are more accurate than single-parameter scores, such as those determined with BTF.^5,23,24^ Future studies comparing inpatient deterioration risk scores may use the NEWS or MEWS as robust comparators when assessing incremental gains in model performance.

Third, discrimination for the combined outcome of ICU transfer or death was only poor to adequate for all of the points-based scores that we evaluated. This finding is a key consideration because the goal of early warning systems is to improve the identification of high-risk patients and enable clinical interventions that can mitigate or prevent deterioration, including proactive transfer to the ICU. Several studies suggested that more advanced regression- or machine learning–based risk scores improve model performance in this setting.^1,2,25^ These tools, including the Advance Alert Monitor and eCART scores, can increase alert accuracy while also decreasing the number needed-to-screen ratios by leveraging more granular EHR data with the additional benefit of seamless calculation.^1,2,26^ All scores demonstrated worsened performance in the non-infected subgroup, suggesting that more-advanced machine learning–based risk scores may be needed to improve the performance and utility of risk scoring in this population. Again, depending on the setting, implementation of these significantly more complex machine learning models may not be feasible or efficient.

### Strengths and Limitations 

The major strength of this study is its use of a large cohort of hospitalized patients drawn from multiple hospitals and health systems across 2 US states. Another strength is that, despite differences in patient case-mix, locale, practice, and teaching status, our results were similar across the states. Although there were differences in the proportion of patients with infection admitted through the ED between states, we nonetheless identified an apparently consistent level of model performance.

There are also several important limitations. First, the study was conducted in US health centers with robust EHR systems, which may lessen the generalizability of our findings. However, our results are largely consistent with those reported in smaller studies in diverse settings.^11,12,20^ Individual hospitals should attempt their own local evaluation of risk scores before implementation wherever possible. The use of EHR data may also be limited by missing data resulting from incomplete documentation of certain clinical observations, such as altered mental status. Second, we evaluated the performance of maximal early warning scores across a patient’s entire time-at-risk interval rather than evaluating model performance across a specific outcome interval (eg, death within the next 24 hours). Although different time horizons and methods of evaluating performance for dynamic scores may alter the particular AUC point estimates, previous work has suggested that this variability does not affect the ordering of early warning score accuracy.^1,27^ In addition, most patients were assigned a score value that crossed an alerting threshold at least 5 hours before the combined outcome and several days before death. Third, we used the Sepsis-3 definition of infection, which could limit the proportion of eligible hospitalizations considered as infectious. However, a previous study noted that alternative infection or sepsis definitions have a minor association with the discrimination and ordering of model risk score performance.^28^ Fourth, our outcome included death and/or ICU transfer, which had the potential of including expected hospital deaths as well as ICU transfers that did not reflect actual clinical deterioration (ie, elective transfers). While this composite outcome might reduce the clinical utility of risk scores in practice, it would be unlikely to influence the overall ordering of model discrimination.^29^

Fifth, the ability to more accurately identify patients at high risk does not necessarily translate into improved outcomes. While the past decade has seen great interest in using complex, large-scale health data to improve risk prediction with a rapidly expanding number of early warning systems, few studies have noted that these advanced tools improve patient outcomes, particularly when patients are randomized and compared with standard care or simpler point-based scoring systems.^27^ Given the substantial challenges that can accompany tool implementation, investment is needed in not only selecting optimal risk scores but in ensuring that the corresponding implementation is effective, efficient, safe, and sustainable.^30^

## Conclusions

In a study spanning 2 states and 28 hospitals, we found that the NEWS appears to be the most efficient points-based risk score for predicting mortality and ICU transfer in patients outside the ICU. This finding was noted for patients with and without suspected infection. Using a single tool that provides a points-based risk score, such as the NEWS, may improve the integration, training, and deployment of early warning scores into clinical pathways focused on identifying and treating patients at risk for deterioration.

## Notes

Supplement. **eTable 1.** Data Elements and Points Associated With Each of the Five Risk Scores Included in This Study **eTable 2.** Missingness Rates of Data Elements for Calculating Risk Scores Between California and Illinois Hospitals **eTable 3.** Median and Interquartile Range Values for Maximum Risk Score Values During the Time-at-Risk Interval, Stratified by Region and Suspected Infection
