# Supplementary material for: Comparison of Early Warning Scoring Systems for Hospitalized Patients With and Without Infection at Risk for In-Hospital Mortality and Transfer to the Intensive Care Unit
Source: JAMA Netw Open. 2020 May 19;3(5):e205191. doi: 10.1001/jamanetworkopen.2020.5191 (PMC7237982; doi:10.1001/jamanetworkopen.2020.5191)
Supplement: Supplement. — eTable 1. Data Elements and Points Associated With Each of the Five Risk Scores Included in This Study eTable 2. Missingness Rates of Data Elements for Calculating Risk Scores Between California and Illinois Hospitals eTable 3. Median and Interquartile Range Values for Maximum Risk Score Values During the Time-at-Risk Interval, Stratified by Region and Suspected Infection [file jamanetwopen-3-e205191-s001.pdf]

Supplementary Online Content

Liu VX, Lu Y, Carey KA, et al. Comparison of early warning scoring systems for hospitalized patients with and without infection at risk for in-hospital mortality and transfer to the intensive care unit. *JAMA Netw Open*. 2020;3(5):e205191. doi:10.1001/jamanetworkopen.2020.5191

eTable 1. Data Elements and Points Associated With Each of the Five Risk Scores Included in This Study

eTable 2. Missingness Rates of Data Elements for Calculating Risk Scores Between California and Illinois Hospitals

eTable 3. Median and Interquartile Range Values for Maximum Risk Score Values During the Time-at-Risk Interval, Stratified by Region and Suspected Infection

This supplementary material has been provided by the authors to give readers additional information about their work.

© 2020 Liu VX et al. *JAMA Network Open.*

eTable 1. Data elements and points associated with each of the five risk scores included in this study.

NEWS *(score range: 0-20)*	***MEWS*** *(score range: 0-14)*	***BTF**** *(score range: Yellow or red)*	***SIRS*** *(score range: 0-4)*	***qSOFA*** *(score range: 0-3)*
**RR** (0-3)	**RR** (0-3)	**RR** (yellow/red)	**RR** (0 or 1)	**RR** (0 or 1)
**Temp (°C)** (0-3)	**Temp (°C)** (0-2)	**Temp (°C)** (yellow/red)	**Temp (°C)** (0 or 1)	
**Systolic BP** (0-3)	**Systolic BP** (0-3)	**Systolic BP** (yellow/red)		**Systolic BP** (0 or 1)
**HR** (0-3)	**HR** (0-3)	**HR** (yellow/red)	**HR** (0 or 1)	
**AVPU** (0 or 3)	**AVPU** (0-3)	**AVPU** (yellow/red)		**GCS** (0 or 1)
**Sp02 (%)** (0-3)		**Sp02 (%)** (yellow/red)		
**Supplemental oxygen** (0 or 2)				
			**WBC** (0 or 1)	

*any parameter may qualify the patient to be in yellow or red zone and is a binary score
NEW = National Early Warning Score; MEWS = Modified Early Warning Score; BTF = Between the Flages; SIRS = Systemic Inflammatory Respons Syndrome; qSOFA = quick Sequential Organ Failure Assessment; RR = respiratory rate (breaths); Sp02 = peripheral capillary oxygen saturation percentage; Temp (celcius) = temperature; BP = blood pressure (mmHg); AVPU = alert, voice, pain, unresponsive; GCS = Glasgow coma scale; WBC = white blood cell count (mm^3); BUN = blood urea nitrogen (mg/dL or mmol/L)

© 2020 Liu VX et al. *JAMA Network Open.*

eTable 2. Missingness rates of data elements for calculating risk scores between California and Illinois hospitals.

**Variable**	**California hospitals**	**Illinois hospitals**
Heart rate	910 (0%)	1,593 (0%)
Respiratory rate	1,347 (0%)	1,630 (0%)
Systolic blood pressure	977 (0%)	1,817 (0%)
Diastolic blood pressure	977 (0%)	1,830 (0%)
Oxygen saturation	1328 (0%)	8,523 (1%)
Temperature	4,491 (0%)	3,924 (1%)
Neurologic evaluation*	460,274 (47%)	22,449 (3%)
Glasgow coma score	460,481 (47%)	289,614 (41%)
Band forms	855,150 (87%)	297,666 (42%)
Blood urea nitrogen	108,824 (11%)	85,010 (12%)
White blood cell count	69,929 (7%)	63,405 (9%)

* Neurologic evaluation in California hospitals based on identification of only abnormal neurologic status based on flowsheet documentation from electronic health record data, as described in *Escobar GJ, Gardner MN, Greene JD, Draper D, Kipnis P. Risk-adjusting hospital mortality using a comprehensive electronic record in an integrated health care delivery system. Medical Care 2013; 6(2): 74-80.*

© 2020 Liu VX et al. *JAMA Network Open.*

eTable 3. Median and interquartile range values for maximum risk score values during the time-at-risk interval, stratified by region and suspected infection.

	**California hospitals** *(n = 752,130)*		**Illinois hospitals** *(n = 713,786)*	
	**Suspected Infection**			
	Yes	***No***	***Yes***	***No***
**General**				
NEWS	6 (4 – 8)	5 (3 – 6)	6 (4 – 8)	4 (3 – 6)
MEWS	3 (2 – 4)	2 (1 – 3)	3 (2 – 5)	2 (2 – 3)
BTF	2 (1 – 2)	1 (1 – 2)	2 (1 – 2)	1 (1 – 2)
**Infection-related**				
qSOFA	1 (1 – 2)	1 (0 – 1)	1 (1 – 1)	1 (0 – 1)
SIRS	2 (1 – 3)	1 (1 – 2)	2 (1 – 3)	1 (1 – 2)

Abbreviations: NEWS: National Early Warning Score; MEWS: Modified Early Warning Score; BTF: Between the Flags; qSOFA: quick Sepsis-related Organ Failure Assessment; SIRS: Systemic Inflammatory Response Syndrome

© 2020 Liu VX et al. *JAMA Network Open.*
